# Significant increase in hEGF uptake is correlated with formation of EGFR dimers induced by the EGFR tyrosine kinase inhibitor gefitinib

**DOI:** 10.1007/s00280-013-2198-6

**Published:** 2013-06-08

**Authors:** Peng He, Gang Li

**Affiliations:** Department of Biochemistry and Molecular Biology, Peking University Health Science Center, Beijing, 100191 People’s Republic of China

**Keywords:** Gefitinib, hEGF, EGFR, Dimer

## Abstract

**Purpose:**

The epidermal growth factor receptor (EGFR) tyrosine kinase inhibitor gefitinib (ZD1839, Iressa) is approved for cancer treatment. We investigated whether gefitinib treatment can enhance human EGF (hEGF) uptake in vitro, thereby increasing the potential of hEGF as a vehicle for EGFR-targeted therapy.

**Methods:**

Western blotting was used to detect the effect of gefitinib on EGFR signaling. Different EGFR-expressing tumor cells (SCC-1, 22B, A549, and HT-29) were pretreated with gefitinib, and then with ^125^I-hEGF or ^125^I-Vectibix (an anti-EGFR monoclonal antibody). Cell-associated activity was then measured. A cross-linking assay detected increased EGFR dimer formation in gefitinib-treated cells.

**Results:**

Total EGFR levels were not changed, but EGFR phosphorylation was reduced in cells pretreated with gefitinib. Gefitinib mediated formation of EGFR dimers; binding of ^125^I-hEGF to cells pretreated with gefitinib significantly increased. In contrast, binding of ^125^I-Vectibix to tumor cells did not increase. Although total EGFR levels did not increase, binding of hEGF to EGFR + tumors was significantly enhanced after gefitinib treatment, because of increased hEGF binding to gefitinib-induced EGFR dimers.

**Conclusion:**

These results suggest that hEGF could enhance EGFR-targeting when used with gefitinib.

## Introduction

Receptor tyrosine kinases of the epidermal growth factor receptor (EGFR) family are critical in the etiology and progression of solid tumors. EGFR is associated with essential cellular functions, including proliferation, migration, survival, and differentiation [1, 2]; it is over-expressed in many tumors, including head and neck, renal, lung, glioma, breast, colorectal, prostate, ovarian, bladder and pancreas [3], and prompts more aggressive malignant phenotypes and poor prognoses. For such reasons, EGFR is an attractive target for tumor therapies. Gefitinib (ZD1839, Iressa™) is an anilino-quinazoline derivative that acts as an EGFR-specific pharmacological inhibitor; it decreases EGFR kinase activity by binding the adenosine triphosphate (ATP) pocket of the catalytic domain [4]. Although patients with high EGFR expression reportedly have significantly worse survival, gefitinib induces substantial clinical responses in about 10 % of patients with chemotherapy-refractory NSCLC [5–8]. Generally, gefitinib-responsive lung cancers—but not nonresponsive cancers—harbor mutations within the EGFR kinase domain [9, 10]. These heterozygous mutations include small in-frame deletions and missense substitutions clustered within the ATP-binding pocket.

The natural ligand to the EGFR, hEGF, is a peptide with a molecular weight of about 6 kDa. Reportedly, the EGFR-specific TKIs AG-1478 and AG-1517 both compete with ATP in the classical mode of action, and induce formation of inactive, unphosphorylated EGFR dimers, even in the absence of ligand hEGF [11, 12]. The same phenomenon was also observed in cells treated with gefitinib. Gefitinib induces formation of inactive EGFR/HER2 and EGFR/HER3 heterodimers in HER2-overexpressing breast cancer cells [13]. In A431 cells, gefitinib increased the number of EGFR dimers 3.0–3.8 times in the absence of hEGF [14]. In hEGF-stimulated SKOV3 cells, EGFR dimers were also increased 1.8–2.2 times by gefitinib, but this effect was cancelled by pertuzumab [14]. As receptor dimers are thought to have high affinity for their ligand, uptake of ^211^At-hEGF in U343 cells markedly increased (up to 3.5 times) in cells pretreated with gefitinib [15]. Gefitinib treatment increased EGFR B_max_ by membrane stabilization of inactive receptor dimers in the absence of hEGF [16].

Taken together, we supposed that gefitinib induces EGFR dimer formation, thus affecting ligand interactions. This indicates that combined gefitinib treatment and radionuclide targeting to EGFR would be a useful therapeutic modality, even for patients who express high EGFR levels and do not respond to treatment with gefitinib alone or other EGFR-targeted therapeutic methods.

## Materials and methods

### Cell culture

Four cell lines were used: human head and neck squamous carcinoma cell line UM-SCC-22B and SCC1, human colon carcinoma cell line HT29, and human nonsmall cell lung carcinoma cell line A549. All cells were cultured in DMEM supplemented with 10 % fetal bovine serum (FBS) at 37 °C in 5 % CO_2_.

### Radiolabeling

Human epidermal growth factor (hEGF, Chemicon International, USA) and Vectibix (an anti-EGFR mAb, Amgen, Inc.) were labeled with ^125^I (Beijing Atom High Tech, Beijing, China) using the Iodogen method for all cellular experiments. The methods were performed as described previously [17–19].

### Cell-binding assay

Different EGFR-expressing tumor cells seeded in 24-well plates were pretreated with gefitinib at a single dose (400 nM) for 1 h at 4 °C, incubated by addition of ^125^I-hEGF or ^125^I-Vectibix for another 2 h at 4 °C. The total volume of each well was adjusted to 200 μl. Nonspecific binding at each well was determined by adding the same dose of ^125^I-hEGF or ^125^I-Vectibix in the absence of cells. After incubation, the reaction medium was removed and cells were washed with ice-cold PBS. Cells were lysed with 2 M NaOH, and cell-associated radioactivity was measured using a gamma counter (Wallac 1470-002, Perkin-Elmer, Finland). Results were analyzed by GraphPad Prism 4.0 (GraphPad Software, San Diego, CA). Data were expressed as the average of triplicate samples. Cells were administrated with DMSO as vehicle control.

Cells were pretreated with gefitinib at a continuous dose of 0.01, 0.05, 0.1, 0.5, 1, 5, or 10 μM for 1 h at 4 °C, incubated by addition of ^125^I-hEGF for another 2 h at 4 °C. Then, the next steps were performed as described above.

hEGF affinities that were affected by gefitinib were determined by performing competitive displacement studies using ^125^I-hEGF as the radioligand. ^125^I-hEGF was prepared by labeling hEGF with Na^125^I at a high specific activity (RCP = 100 %) according to the method described previously. Briefly, multiscreen DV filter plates were seeded with ∼10^5^ cells in the binding buffer and incubated with gefitinib at a single dose (10 nM) for 1 h at 4 °C. Cells were then washed with ice-cold PBS and incubated with ^125^I-hEGF in the presence of increasing concentrations of hEGF (0–10 μg/ml). After removing the unbound ^125^I-hEGF, hydrophilic PVDF filters were collected and the radioactivity was determined using a gamma counter (Packard, Meriden, CT). All the in vitro experiments were carried out twice with triplicate samples. Nonspecific binding at each well was determined by adding a high concentration of cold hEGF (400 ng/ml) that completely displaced (>95 %) radiolabeled hEGF binding. Cells treated with DMSO were used as control. Results were calculated by nonlinear regression using GraphPad Prism (GraphPad Software, Inc., San Diego, CA), and reported as an average of these samples plus the standard deviation. For comparison purposes, we also evaluated Vectibix using the same method in vitro assay.

### Western blotting

Different EGFR-expressing tumor cells were pretreated with gefitinib at different concentrations in the presence of hEGF (10 μM). Cells were then collected and lysed in cell lysis buffer (50 mM Tris–HCl, pH 7.4, 1 % NP-40, 0.25 % sodium deoxycholate, 150 mM NaCl, 1 mM EGTA, 1 mM Na_3_VO_4_, 1 mM NaF) containing protease inhibitors. After the insoluble part of the lysates was cleared by centrifugation, protein concentrations were determined by the BCA Protein Assay Kit (Pierce). Forty micrograms of proteins was separated by sodium dodecyl sulfate-polyacrylamide gel electrophoresis (SDS-PAGE) and transferred onto a nitrocellulose (NC) membrane. The primary antibodies used for western blot analysis were against EGFR (SC-05, Santa Cruz), and pEGFR (#2231, Cell Signaling). GAPDH was used as loading control. Bands were detected by enhanced chemiluminescent western blotting detection system (GE Healthcare). Films were then scanned by grayscale mode, and the images were opened in ImageJ (http://rsb.info.nih.gov/ij/).

### Cross-linking assay

Before cross-linking, the cells were serum-starved for 24 h in 0.1 % FBS DMEM. Cells were then exposed to gefitinib at concentrations of 0 , 0.01 , 0.05, 0.1, 0.5, 1, 5, or 10 μM (final concentration) for 1 h at 37 °C, followed by incubation with 100 ng/ml hEGF, or without hEGF as positive control. After being washed three times with ice-cold PBS, 1 mM of the cross-linking reagent BS^3^ in cold PBS was added to cells and maintained on ice for 30 min. The reaction was stopped by 500 mM (final concentration) of glycine in PBS for 5 min at 4 °C. Subsequently, cells were washed with ice-cold PBS, and then scraped in lysis buffer as described previously. After centrifugation at 10,000*g* for 15 min at 4 °C, the supernatants were collected and protein concentrations were determined by the BCA Protein Assay Kit (Pierce). Proteins were separated by SDS-PAGE on 6 % gel; western blot analysis was done as described previously. The primary antibodies were against EGFR (SC-05, Santa Cruz). An ROI was drawn on the first lane; the same size/shape ROI was also applied to the other lanes. Each intensity band correlated with the cell-binding data.

### Statistical analysis

Quantitative data were expressed as mean ± SD. Statistical analysis was done by one-way ANOVA and Student’s *t* test. *P* < 0.05 was considered statistically significant (GraphPad Prism 4.0).

## Results

### Effects of gefitinib on EGFR expression and receptor phosphorylation

**Fig. 1:**
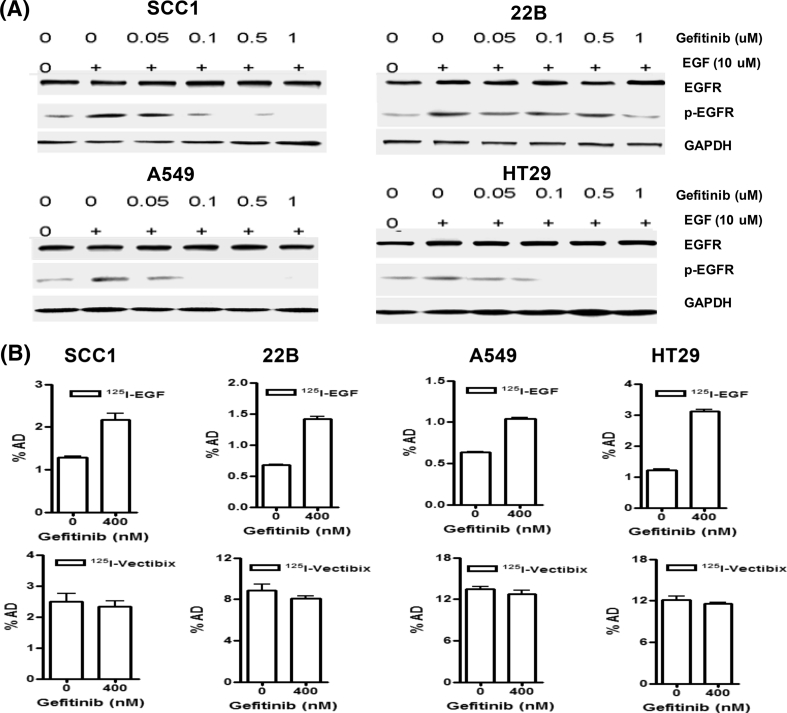
**a** Effects of gefitinib on EGFR expression and receptor phosphorylation in A549, 22B, HT29, and SCC1 cancer cells. **b** Binding of ^125^I-hEGF to cancer cells pretreated with 400 nM gefitinib. The values on the y-axis give the percentage of added radioactivity. Each data point is an average value from three wells ± maximum error

To determine the effects of gefitinib on EGFR expression and receptor phosphorylation, four tumor cells (SCC-1, 22B, A549, and HT-29) were pretreated with gefitinib at 0.00, 0.05, 0.1, 0.5, or 1 μM in the presence of hEGF (10 μM), followed by western blot analysis. As shown in Fig. 1a, these four cell lines showed even levels of EGFR expression, which was not affected by gefitinib, but the phosphorylation of EGFR showed dose-dependent decrease when treated with gefitinib. However, phosphorylation of EGFR in 22B cells did not indicate a dose-dependent decrease.

### Effects of gefitinib on ^125^I-hEGF binding to cells

Cells were pretreated with gefitinib at a single dose (400 nM), followed by addition of ^125^I-hEGF or ^125^I-Vectibix. Binding of ^125^I-hEGF to gefitinib-treated cells was significantly increased, whereas binding of ^125^I-Vectibix to tumor cells did not increase (Fig. 1b).

### Gefitinib mediated formation of EGFR dimer complexes

**Fig. 2:**
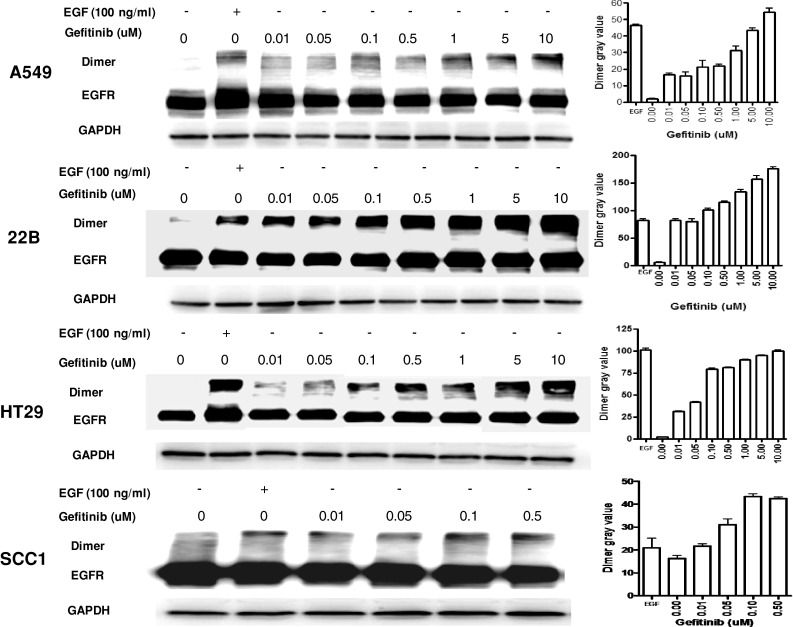
Chemical cross-linking to evaluate EGFR dimerization after gefitinib and/or EGF treatment in A549, 22B, HT29, and SCC1 cancer cells. EGFR dimer formation was evaluated by analysis of gray value

EGFR dimerization is reportedly induced by its specific ligand; it is also mediated by quinazoline derivatives in the absence of growth factor [13]. We verified that this phenomenon appeared in different EGFR-expressing tumor cells (A549, 22B, HT-29, and SCC1) after gefitinib treatment. EGFR dimer formation was detected by western blot experiments, after membrane protein cross-linking, following administration of gefitinib. EGFR monomers (molecular mass 170 kDa) were present in both untreated and drug-treated cells at equal amounts, even at different doses of gefitinib (Fig. 2). However, clear dimerization of EGFR (at ~360 kDa immunoreactive bands) was also evident in the four different tumor cells, and in cells exposed to hEGF (100 ng/ml) treatment; such dimerization gradually increased with the dose of gefitinib at 0.01, 0.05, 0.1, 0.5, 1, 5, and 10 μM (final concentration). Notably, SCC1 cells floated when doses of gefitinib exceeded 0.5 μM, so we only obtained results from 0 to 0.5 μM doses.

### hEGF uptake correlated with increased EGFR dimer formation and change of gefitinib dose

**Fig. 3:**
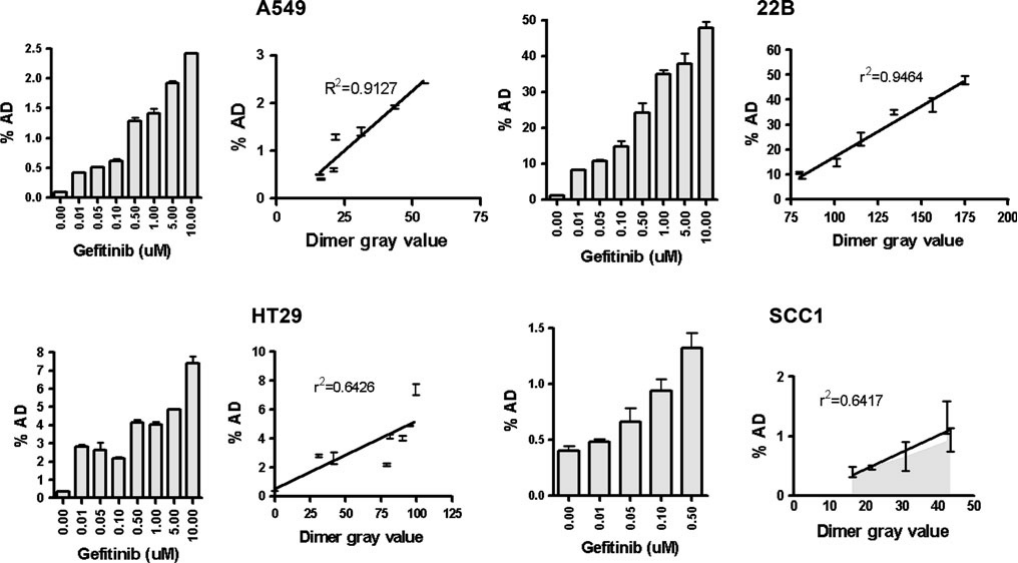
Correlation of hEGF tumor uptake and formation of EGFR dimer dose dependent on gefitinib in A549, 22B, HT29, and SCC1 cancer cells

To determine the correlation between hEGF uptake increase and EGFR dimer formation, we also carried out cell binding of ^125^I-hEGF after gefitinib treatment at continuous doses of 0.01, 0.05, 0.1, 0.5, 1, 5, or 10 μM in cells. We then conducted quantitative densitometry of EGFR dimers, compared the results with those of ^125^I-hEGF cell binding at continuous gefitinib doses, and analyzed the correlation between hEGF uptake increase and EGFR dimer formation. Western blot analysis of EGFR dimers by ImageJ was used to obtain quantitative data (Fig. 3). Relative hEGF uptake was calculated to correlate with the EGFR dimer formation. In all four tumor cell lines, EGFR dimerization and specific binding of hEGF increased with increased gefitinib dose. We therefore supposed that increased hEGF uptake has a better linear correlation of EGFR dimer formation with the change in gefitinib dose, (*r* ^2^ = 0.9127 in A549, *r* ^2^ = 0.9464 in 22B, *r* ^2^ = 0.6426 in HT29, and *r* ^2^ = 0.6417 in SCC1; *P* < 0.0001).

### Gefitinib increased number of hEGF receptor sites

**Fig. 4:**
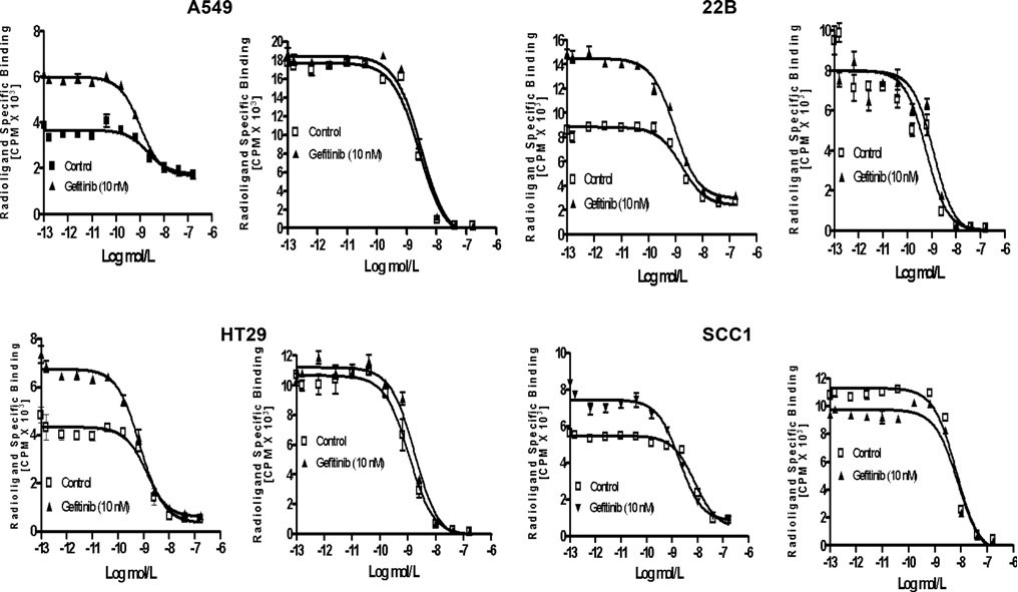
In vitro inhibition of ^125^I-hEGF (*left*) and ^125^I-Vectibix (*right*) binding to EGFR on A549, 22B, HT29, and SCC1 cancer cells by hEGF (*left*) and Vectibix (*right*). *Points* mean (*n* = 3), *bars* SD

The receptor-binding affinity of hEGF treated with gefitinib was determined using competitive displacement studies, with Vectibix as control. hEGF and Vectibix inhibited binding of ^125^I-hEGF and ^125^I-Vectibix to cells, in a concentration-dependent manner (Fig. 4). The results suggest that the gefitinib did not affect the receptor-binding affinities of hEGF and Vectibix. However, hEGF receptor site numbers increased after gefitinib treatment, whereas those for Vectibix did not change, which indicates that gefitinib caused a dramatic increase in radioligand specific binding.

## Discussion

Because of the apparent role of EGFR in tumor aggression and poor prognosis, and its high expression in many types of cancers, the EGFR pathway has been well investigated as a possible target in cancer therapies. Increasing clinical evidence has shown the disparity between the EGFR expression level and the treatment effect of anti-EGFR mAb-based immunotherapeutic agents [20–22]. Epidermal growth factor receptor tyrosine kinase inhibitors (EGFR-TKIs) such as gefitinib represent another strategy for EGFR-targeted cancer therapy. However, gefitinib induces substantial clinical responses in only about 10 % of patients with chemotherapy-refractory NSCLC [5–8]. Besides EGFR expression levels, other factors, such as *EGFR* mutations, tumor microenvironment, tumor vasculature density and permeability, tumor interstitial pressure, pharmacokinetics, and tumor penetration ability of compounds, could influence anti-EGFR treatments [8, 9, 23, 24].

In our study, western blot was performed with different EGFR-expressing tumor cells (SCC-1, 22B, A549, and HT-29) to detect the effects of gefitinib on EGFR expression and receptor phosphorylation. Although the total EGFR levels were unchanged, EGFR phosphorylation was reduced in SCC1, HT29, and A549 cells pretreated with gefitinib (Fig. 1a). However, in 22B cells, EGFR phosphorylation was not decreased in a significant dose-dependent manner when treated with gefitinib, which suggests that the 22B tumor cells are tolerant to gefitinib. When ^125^I-hEGF was added to cells pretreated with gefitinib, an unexpected two–threefold increase was observed (Fig. 1b). Furthermore, the increase was dependent on the drug concentration (Fig. 3). However, ^125^I-Vectibix did not show the same increase (Fig. 1b). Our results indicate that hEGF uptake was significantly increased after gefitinib treatment in EGFR + cells, even in the cells tolerant to gefitinib.

hEGF can induce transphosphorylation of their receptors, mainly by forming monomeric receptors into active dimers. However, quinazoline drugs can induce sequestration of EGFR into inactive dimers [25], and trap the ligand into those complexes, which can block the ligand from binding to still-functional receptors. This pattern is consistent with gefitinib directly promoting formation of inactive EGFR-based homodimers or heterodimers (EGFR/HER2, EGFR/HER3) in several breast carcinoma cell lines [13]. Gefitinib is reported to competitively inhibit ATP binding at the catalytic site of EGFR tyrosine kinase [26]. Some studies showed that gefitinib can cause complete and long-lasting inhibition of EGFR phosphorylation, which depends on sequestration of inactive drug–receptor complexes in breast and ovarian carcinomas [13, 27]. Interaction of anilino-quinazoline-induced receptor dimerization at the ATP-binding site has been reported even in the absence of ligand binding [11]. Therefore, we hypothesized that the increased hEGF uptake may be due to gefitinib-induced formation of inactive dimers, and such dimers could be responsible for the apparent increase in EGFR binding sites. We confirmed significantly increased EGFR dimerization after gefitinib treatment, which gradually increased with gefitinib dose (Fig. 2). An even better linear correlation between hEGF uptake increase and EGFR dimer formation was seen as gefitinib dose increased (Fig. 3). Additionally, the dimers stabilized in the nonphosphorylated (inactive) state in the presence of hEGF (data not showed), as previously reported [14]. Thus, stabilization of inactive dimers may represent another way by which gefitinib impairs EGFR activity. The competitive cell-binding assay showed that the affinity of EGFR was not significantly changed after gefitinib treatment, but the hEGF receptor site numbers (but not those for Vectibix) were increased after gefitinib treatment (Fig. 4). It may be hypothesized that either the gefitinib-dependent EGFR dimerization exposed new binding sites in the inactivated receptors or that dimerization allows hEGF access to spare receptors sequestered in membrane compartments that prevent ligand binding in untreated cells.

Although our previous study [25] independently demonstrates that formation of nonfunctional EGFR dimers can be induced by gefitinib, the exact molecular mechanism linking both events is unclear. However, this study is the first to report the correlation between increased ^125^I-hEGF and EGFR dimer formation with changes in gefitinib dose.

In summary, the main goal of this study was to establish a basis for investigating the mechanism of gefitinib-mediated increase in hEGF uptake. Although total EGFR levels did not increase, the binding of hEGF to EGFR + tumors was significantly enhanced after gefitinib treatment, which is likely due to hEGF binding to increased numbers of gefitinib-induced EGFR dimers. These results suggest that hEGF could enhance EGFR-targeting when used with gefitinib.
